# Unraveling Complexity: Acute Intermittent Porphyria Complicated by Rhabdomyolysis and Acute Pancreatitis

**DOI:** 10.7759/cureus.65826

**Published:** 2024-07-31

**Authors:** Joseph Norman, Mario Soliman

**Affiliations:** 1 Medicine, A.T. Still University of Health Sciences-Kirksville College of Osteopathic Medicine, Kirksville, USA; 2 Family Medicine, University of Pittsburgh Medical Center Pinnacle, Lititz, USA

**Keywords:** guillain-barré syndrome (gbs), acute intermittent porphyria, acute porphyria management, rhabdomyolysis, pancreatitis, acute porphyria symptoms

## Abstract

Acute intermittent porphyria (AIP) is a rare autosomal dominant disorder characterized by defective porphyrin metabolism in the blood. It manifests through variable clinical features, among these are abdominal pain, nausea, vomiting, peripheral neuropathy, and seizure. The diverse presentation of AIP poses substantial diagnostic challenges due to its potential to mimic other medical conditions, delaying early recognition and intervention. Management strategies of AIP involve a multifaceted approach, focusing on symptom relief and attack cessation. Early recognition and intervention are pivotal in optimizing patient outcomes, highlighting the importance of heightened clinical suspicion and precise diagnostic pathways.

We present a unique case of a 34-year-old female who presented to the emergency department with severe abdominal pain, oliguria, and progressive sensory and motor deficits. Despite exhibiting hallmark symptoms suggestive of AIP, the absence of distinctive "attack periods" added complexity to the diagnostic process, requiring the exclusion of other medical conditions with similar overlapping symptoms.

## Introduction

Porphyria describes a group of conditions that disrupt the heme synthesis pathway, usually related to genetic enzyme deficiency. Acute intermittent porphyria (AIP) is a type of porphyria caused by an autosomal dominant gene mutation in the porphobilinogen deaminase enzyme (PBGD), creating an excess of pathway intermediates that cause a difficult-to-recognize mosaic of symptoms for brief periods termed attacks [1].

Based on case series, the most common of these symptoms are abdominal pain (85-95%), vomiting (43-88%), constipation (48-84%), muscle pains (50-70%), paresis (42-68%), tachycardia (64-85%), and psychiatric symptoms such as confusion (50-70%) [2]. As the inheritance of the PBGD gene mutation is not sufficient to cause attacks, investigation of other aggravating factors has correlated nutritional deficiency, alcohol, smoking, and use of drugs that interfere with the heme metabolic pathway. Female sex is also a risk factor due to menstrual fluctuation in androgenic hormones, as such hormones act as CYP enzyme substrates [3,4]. We report a unique case of AIP in a symptomatic 34-year-old female, presenting in an emergency department (ED) setting, with a complicated past and current medical history.

## Case presentation

A 34-year-old woman presented to the ED with two months of intermittent abdominal pain and nausea accompanied by new-onset facial numbness and weakness in her right upper and left lower extremities. She has a past medical history of anxiety, bipolar disorder, reflux disease, deep vein thrombosis, and a prior episode of Guillain-Barré syndrome (GBS). During this episode, the patient had experienced ascending weakness in the feet and ankles, which resolved gradually with intravenous (IV) immunoglobulin therapy. In collecting social history, she reported significant smoking history, alcohol use, and prior IV drug abuse, primarily heroin, but attested abstinence for two years. She had three prior cesarean sections and was on topiramate, lamotrigine, gabapentin, and metoprolol, along with an etonogestrel subdermal implant.

In the ED, the patient was afebrile and tachycardic with a heart rate of 132, a blood pressure of 135/108 mmHg, a respiratory rate of 18, and an oxygen saturation of 100% on room air. The physical exam revealed an ill-appearing patient with right lower quadrant abdominal tenderness to palpation without rebound or guarding. The neurological exam revealed right-sided facial paresthesia, right upper extremity 0/5 strength testing with paresthesia in the C5-T1 dermatomal distribution, left lower extremity 0/5 strength testing, and 5/5 strength in the left upper and right lower extremities.

**Figure 1:**
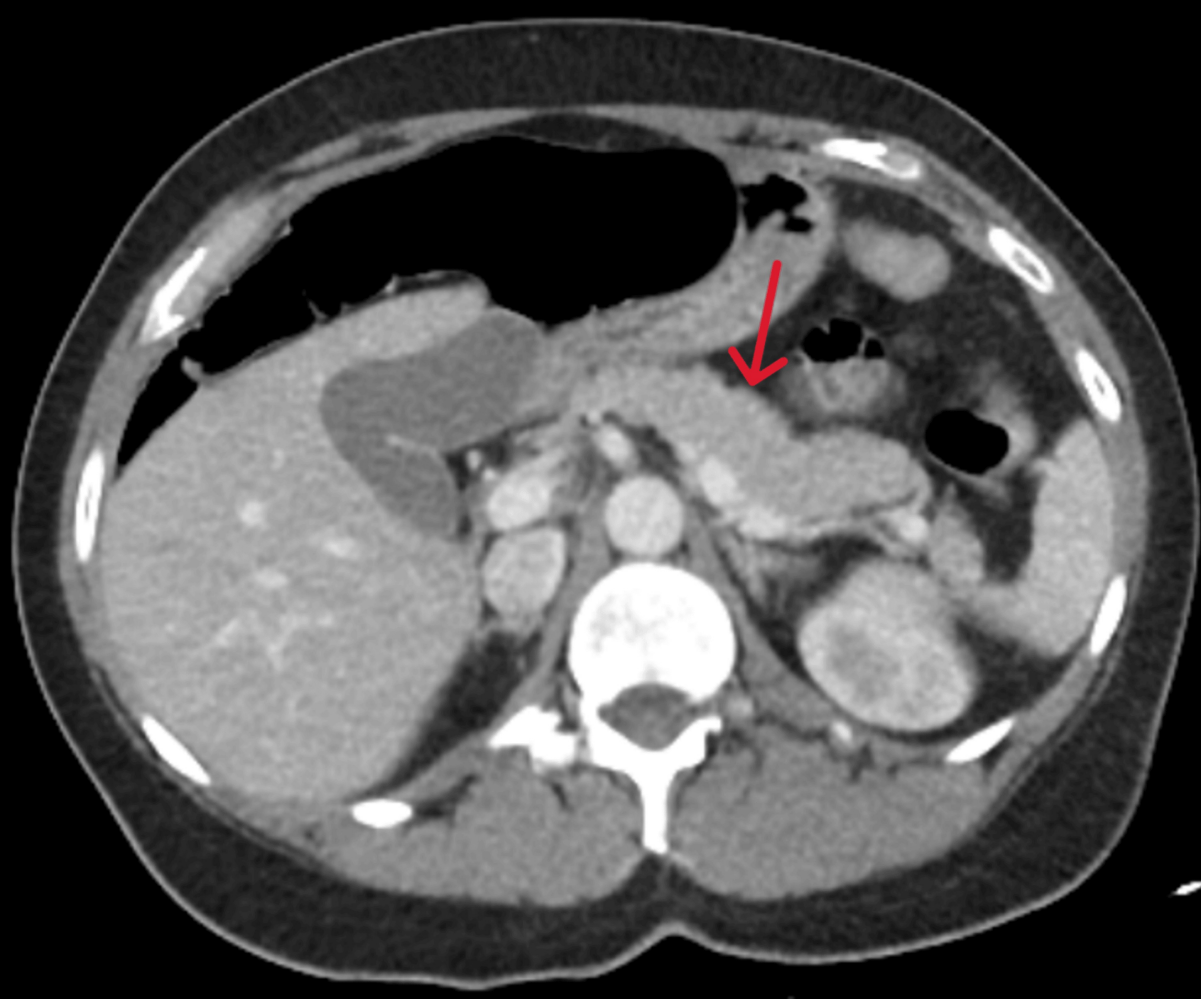
Axial abdominal CT CT scan of the abdomen and pelvis shows mild diffuse pancreatic parenchymal swelling with minimal changes in density suggestive of mild pancreatitis. No evidence of necrosis, pseudocysts, or abscesses in the pancreas or surrounding tissues. Otherwise unremarkable CT of the abdomen and pelvis. CT: computed tomography

The ED workup was notable for elevated absolute neutrophilia of 15.1 k/microL, hyponatremia with a serum sodium level of 127 mmol/L, elevated lipase at 343 U/L, elevated creatine kinase at 1,393 U/L, elevated creatinine level of 2.58 mg/dl, and transaminitis with elevated aspartate aminotransferase at 77 U/L and alanine aminotransferase at 63 U/L. The erythrocyte sedimentation rate was 27 mm/hr, and the alkaline phosphatase was 63 U/L (Table 1). The urine drug screen was negative for all tested substances. The serum pregnancy test was negative. High-sensitivity troponins, lactic acid, and beta-hydroxybutyrate were all within normal limits. Urinalysis was notable for brown cloudy urine with a specific gravity of 1.030, moderate bilirubin, trace blood, 1+ protein, 1+ leukocyte esterase, and 4.0 mg/dl urobilinogen (Table 2). Abdominal and pelvis computed tomography (CT) was suggestive of mild pancreatitis (Figure 1). Brain magnetic resonance imaging (MRI) was largely unremarkable.

**Table 1:** Summary of laboratory tests conducted, including patient values and reference ranges

Laboratory tests	Patient's value	Normal range
White blood cell count	15,100/ul	1,800-7,400/ul
Serum sodium	127 mmol/L	135-145 mmol/L
Serum lipase	343 U/L	11-82 U/L
Creatine kinase	1,393 U/L	38-234 U/L
Creatinine	2.58 mg/dl	0.6-1.2 mg/dl
Aspartate aminotransferase	77 U/L	8-33 U/L
Alanine aminotransferase	63 U/L	4-36 U/L
Erythrocyte sedimentation rate	27 mm/hour	0-20 mm/hour
Alkaline phosphatase	63 U/L	23-127 U/L

**Table 2:** Urinalysis testing conducted, including patient values and reference ranges

Urine testing	Patient's value	Normal range
Urine-specific gravity	1.030	1.005-1.030
Urine protein	30 mg/dl	<10 mg/dl
Leukocyte esterase	1+	Negative
Urobilinogen	4.0 mg/dl	0.1-1.8 mg/dl

The patient was admitted for the evaluation and treatment of acute pancreatitis, acute kidney injury, and possible rhabdomyolysis. She received IV fluids as well as pain management. Antibiotics were started due to the systemic inflammatory response syndrome (SIRS) criteria. Consultations were made with surgical, gastroenterology, and neurology teams. Daily neurological examination did not show any significant changes, prompting cerebrospinal fluid (CSF) testing and spinal imaging to investigate multiple sclerosis, both of which were unremarkable.

On day 3 of hospitalization, grossly dark/wine-colored urine output was observed. Given the prior reported episode of GBS in the setting of concurrent presentation of abdominal pain, increased urobilinogen on initial urinalysis, and neurological symptoms, a porphyria workup was initiated. Urine testing revealed elevated porphyrins suggesting the diagnosis of AIP. Due to clinical improvement, the patient declined specific treatment for porphyria and was discharged home with a recommended follow-up with hematology for porphyria management as well as genetic counseling.

Outpatient testing of 24-hour urine porphobilinogen testing with quantitative porphyrin fractions revealed marked elevation in uroporphyrin I at 499.3 mcg and uroporphyrin III at 190.4 mcg. Reference ranges and other heme metabolite products collected in testing can be found in Table 3.

**Table 3:** 24-hour quantitative urine porphyrin patient values and reference ranges

Urine porphyrin testing (24 hours)	Patient's value	Normal range
Uroporphyrin I	499.3 mcg	4.1-22.4 mcg/24 h
Uroporphyrin III	190.4 mcg	0.7-7.4 mcg/24 h
Heptacarboxylporphyrin	55.4 mcg	≤3.3 mcg/24 h
Hexacarboxylporphyrin	2.4 mcg	≤10 mcg/24 h
Pentacarboxylporphyrin	24.4 mcg	≤4.6 mcg/24 h
Coproporphyrin I	62 mcg	7.1-48.7 mcg/24 h
Total porphyrin count	1049.3 mcg	35.0-210.7 mcg/24 h

These findings conclusively confirmed the diagnosis of AIP. The patient was later evaluated by hematology for comprehensive counseling regarding the management of acute attacks, genetic testing, and strategies for prevention through risk factor mitigation.

## Discussion

The clinical manifestation of AIP often poses a diagnostic challenge, with a delayed time to diagnosis and a propensity for recurrent attacks prior to proper diagnosis [5,6]. Our patient presented with abdominal pain, a common symptom of AIP, albeit not confined to discrete "attack periods" as commonly reported [1]. A recent study highlighted chronic abdominal pain as a notable feature in some AIP patients, impacting diet and accompanied by nausea, neuropathic symptoms, and musculoskeletal pain [6]. Our patient reported baseline abdominal pain with exacerbations over two months, managing associated nausea and vomiting through dietary restriction. Additionally, she reported a prior episode of GBS with unresolved neurological symptoms despite treatment. The patient's recent-onset facial weakness and subsequent extremity weakness were of immediate concern and initially raised suspicion of a recurrent GBS episode, given her prior medical history. Interestingly, GBS has been noted in several reports as a pitfall diagnosis to AIP due to the shared presentation of rapidly progressing limb weakness [7,8]. However, other differential diagnoses, including cerebrovascular events, illicit substance ingestion, multiple sclerosis, and electrolyte abnormalities, needed consideration. Radiological and laboratory investigations were pursued to explore these possibilities. 

Despite inconclusive abdominal CT imaging, our patient's laboratory studies were most notable for lipase levels that are three times the upper limit of normal (Table 1); this strongly suggests acute pancreatitis for which the patient received aggressive IV fluid hydration and adequate pain regimen. Although there is no direct link between acute pancreatitis and AIP, there have been case reports of acute pancreatitis complications in the setting of AIP [9-11]. Follow-up studies, including brain MRI, spinal MRI, and CSF testing, aimed to exclude other neurological conditions such as GBS, multiple sclerosis, and transverse myelitis. However, the patient's clinical status remained largely unchanged, except for some pain relief with analgesic therapy.

Our patient also presented with initially elevated creatine kinase, hyponatremia, and sensory symptoms that initially suggested a rhabdomyolysis picture; this was an alternative explanation for the dark/wine-colored urine. This illustrates the diagnostic challenges of AIP and its overlapping symptoms with rhabdomyolysis. This has been shown in several case reports [12,13].

Although it is classified as a genetic condition, AIP exhibits a difficult-to-predict pattern of attack occurrence due to the extremely low penetrance for PBGD mutations of approximately 1% [14]. As such, it is necessary to identify environmental factors that may precipitate or increase the frequency of attacks. Known environmental risk factors contributing to AIP attacks include alcohol consumption, fasting or caloric restrictions, smoking, medications that inhibit or induce certain cytochrome P450 enzymes, and endogenous steroid hormones [2]. Our patient reported dietary restrictions, a longstanding history of cigarette smoking, alcohol use, as well as recent etonogestrel implantation three weeks prior to the first documented encounter for her abdominal symptoms. A recent report described a patient case of AIP that was precipitated with the recent implantation of etonogestrel [15]. Interestingly, etonogestrel is listed on the Norwegian Porphyria Centre (NAPOS)'s drug database as a "probably porphyrinogenic" medication as it inhibits certain cytochrome P450 enzymes [16].

AIP is diagnosed with 24-hour urine porphobilinogen testing and quantitative porphyrin fractions; our patient's results revealed marked elevation in uroporphyrin I at 499.3 mcg and uroporphyrin III at 190.4 mcg indicating AIP from among other types of acute porphyrias [17].

Treatment strategies for AIP involve symptomatic management and attack cessation therapies. In managing pain for AIP patients, it is recommended to use acetaminophen and opioid analgesics while avoiding nonsteroidal anti-inflammatory drugs (NSAIDs) to prevent exacerbation of attack [18]. Early hematin can aid in attack cessation and works to upregulate the excretion of aminolevulinic acid (ALA) and porphobilinogen (PBG), for rapid clinical improvement and halting the progression of neuropathy. It is given at 2-4 mg/kg/day for 4-14 days and has a low side effect profile with short-term use [18]. Glucose-rich dietary options or IV administration of up to 300 g of dextrose using a 10% dextrose solution can also contribute to halting an attack by inhibiting aminolevulinic acid synthase (ALAS) [2,18]. In recent developments, the ENVISION phase 3 clinical trial evaluating givosiran, an RNA inhibitor targeting ALAS protein translation, exhibited promising results, demonstrating a notable 74% decrease in the annual occurrence of AIP attacks within a cohort of 94 patients [19]. However, it is noteworthy that a heightened prevalence of chronic kidney disease and elevated aminotransferase enzymes was observed among participants receiving givosiran [19]. Liver transplantation remains a final recourse in managing severe and enduring AIP attacks, showing efficacy in certain cases [20].

## Conclusions

This case report underscores the complexity and diagnostic challenges associated with AIP and its variable presentation delaying diagnosis while attacks recur. Our patient's case highlights the importance of considering AIP in patients with abdominal pain and neurological symptoms, even in the absence of classic "attack periods." Diagnosis of AIP may require differentiation from GBS, acute pancreatitis, rhabdomyolysis, and multiple sclerosis. Despite these challenges, a thorough assessment and confirmatory urinary porphyrin testing led to the diagnosis of AIP in our patient. Early recognition and appropriate management are crucial in managing symptoms, preventing complications, and improving outcomes.
